# The ethical requirement of explainability for AI-DSS in healthcare: a systematic review of reasons

**DOI:** 10.1186/s12910-024-01103-2

**Published:** 2024-10-01

**Authors:** Nils Freyer, Dominik Groß, Myriam Lipprandt

**Affiliations:** 1 Institute of Medical Informatics, Medical Faculty, RWTH Aachen University, Aachen, Germany; 2 Institute for the History, Theory and Ethics of Medicine, Medical Faculty, RWTH Aachen University, Aachen, Germany

**Keywords:** Decision-support systems, Artificial Intelligence, Systematic-review, Explainability, Transparency, Explicability, Healthcare

## Abstract

**Background:**

Despite continuous performance improvements, especially in clinical contexts, a major challenge of Artificial Intelligence based Decision Support Systems (AI-DSS) remains their degree of epistemic opacity. The conditions of and the solutions for the justified use of the occasionally unexplainable technology in healthcare are an active field of research. In March 2024, the European Union agreed upon the Artificial Intelligence Act (AIA), requiring medical AI-DSS to be ad-hoc explainable or to use post-hoc explainability methods. The ethical debate does not seem to settle on this requirement yet. This systematic review aims to outline and categorize the positions and arguments in the ethical debate.

**Methods:**

We conducted a literature search on PubMed, BASE, and Scopus for English-speaking scientific peer-reviewed publications from 2016 to 2024. The inclusion criterion was to give explicit requirements of explainability for AI-DSS in healthcare and reason for it. Non-domain-specific documents, as well as surveys, reviews, and meta-analyses were excluded. The ethical requirements for explainability outlined in the documents were qualitatively analyzed with respect to arguments for the requirement of explainability and the required level of explainability.

**Results:**

The literature search resulted in 1662 documents; 44 documents were included in the review after eligibility screening of the remaining full texts. Our analysis showed that 17 records argue in favor of the requirement of explainable AI methods (xAI) or ad-hoc explainable models, providing 9 categories of arguments. The other 27 records argued against a general requirement, providing 11 categories of arguments. Also, we found that 14 works advocate the need for context-dependent levels of explainability, as opposed to 30 documents, arguing for context-independent, absolute standards.

**Conclusions:**

The systematic review of reasons shows no clear agreement on the requirement of post-hoc explainability methods or ad-hoc explainable models for AI-DSS in healthcare. The arguments found in the debate were referenced and responded to from different perspectives, demonstrating an interactive discourse. Policymakers and researchers should watch the development of the debate closely. Conversely, ethicists should be well informed by empirical and technical research, given the frequency of advancements in the field.

**Supplementary Information:**

The online version contains supplementary material available at 10.1186/s12910-024-01103-2.

## Background

Using Artificial Intelligence based Decision Support Systems (AI-DSS) in healthcare applications appears valuable by increasing accessibility, precision, and speed of medical decision-making [1, 2] – provided that their use is critically reflected upon. Healthcare professionals (HCPs) with limited clinical experience in particular can benefit from AI-DSS. While AI-DSS originate from symbolic AI and rule-based approaches to decision support, contemporary approaches to such systems are mostly based on machine learning algorithms and, more specifically, on deep learning techniques. Deep-learning-based AI-DSS impress with performance [3]. Currently, the impression is growing that the technical development of AI-DSS and the definition of the appropriate ethical framework for the use of this technology are increasingly detached from each other – a phenomenon also known as the Collingridge dilemma (after David Collingridge). It describes a methodological dilemma in which efforts to influence or control the further development of the technology are confronted with a double-bind problem: An information problem: the effects cannot be readily predicted until the technology is widely developed and disseminated. And a power problem: control or change is difficult once the technology has become established [4].

In other words: AI-DSS introduced their own line of both ethical and regulatory concerns. To address this, the European Union introduced the first regulatory framework on AI, coming into force in 2024: the Artificial Intelligence Act (AIA). The AIA is supposed to address, among others, concerns of trustworthiness, human rights, and explainability [5, 6]. In fact, a major concern that is often taken to be specific to AI-DSS is one of explainability, i.e., their level of epistemic^1^ opacity. In short, epistemic opacity denotes the inaccessibility of the processes and attributes of a computational system (cf. Terminology and [7]). While formerly mostly a matter of debate for the philosophy of science, the ethical debate around this concern most famously took off with the AI4People framework by Floridi et al. in 2018, introducing the principle of explicability in the AI-ethics context [8].

The AIA defines all medical devices as defined in the medical device regulation or the in-vitro medical device regulation as ‘high-risk’ systems (cf. Annex II of the AIA [6] and Gilbert’s analysis [9]). Such high-risk systems impose distinctive requirements that address their epistemic opacity. In AIA Art. 13 3. (d), human oversight measures are required for high-risk AI systems, including the ability to.

- “[…] correctly interpret the high-risk AI system’s output, taking into account in particular the characteristics of the system and the interpretation tools and methods available” (Art. 14 4. (c)), and.
- “[…] fully understand the capacities and limitations of the high-risk AI system and be able to duly monitor its operation, so that signs of anomalies, dysfunctions, and unexpected performance can be detected and addressed as soon as possible;” (Art. 14. 4. (a)) [6].

Therefore, by the AIA, AI-DSS need to either implement explainable Artificial Intelligence (xAI, post-hoc) methods or use intrinsically explainable (ad-hoc) models in the first place. At the same time, the epistemic opacity of deep learning techniques motivates a vast and ongoing debate on the ethical permissibility of AI-DSS in healthcare, characterized by terminological incoherences across disciplines [10, 11]. The normative standards of explainability denote the specifications the AI-DSS must satisfy to be ethically acceptable.

To outline the ethical debate and find arguments in favor of and against the requirements of the AIA, we conducted a systematic review of reasons [12], complying with the PRISMA standards [13, 14] (cf. Section 2).

In this systematic review, we will first introduce a terminological common ground and second examine the research objective: *Do the normative standards of explainability for AI-DSS require the use of xAI or ad-hoc explainable models to be ethically acceptable in healthcare?* And *how are the different positions argued for?*

Additionally, we provide a brief overview of the levels of explainability required by the normative standards found in the literature.

## Methods

We conducted a systematic review of reasons to characterize key presumptions and motivations in the debate and to identify the normative standards of explainability according to the PRISMA standards [13, 14]. Therefore, we performed a systematic literature search for reproducible results in April 2024. The literature databases used are the Bielefeld Academic Search Engine (BASE), PubMed, and Scopus, as they cover the relevant domains and offer transparent and reproducible search options and results [15].

### Review design

Due to the recency of the use of AI-DSS in healthcare, the research field of xAI, and the ethical debate emerging from it, we limited our literature search to the period 2016–2024. We designed a search string to include English-speaking, scientifically peer-reviewed literature. The publications searched for were supposed to discuss the ethical permissibility of AI-DSS regarding explainability and its hyponyms and synonyms as one research objective. For the explicit queries for all three databases, cf. Appendix A.

- *W={Ethics, Normative Standards, Normativity}*
- *X={Explainability, Explicability, Interpretability, Contestability, Transparency}*
- *Y={Health, Healthcare, Medicine}*
- *Z={Machine Learning, Artificial Intelligence, Deep Learning}*
- *(w*∧*x*∧*y*∧*z) for w, x, y, z*∈*W×X×Y×Z*

We excluded literature reviews and surveys. This type of publication typically summarizes arguments and positions that we expect to cover by our own analysis of the literature. The exclusion of literature reviews and surveys is supposed to avoid anticipatable duplicates and overrepresentations. Furthermore, we excluded technical and empirical literature that only implicitly or briefly touched ethical questions. While empirical surveys, e.g. on the acceptance of AI-DSS in healthcare, may bring up arguments and positions from important stakeholders, we limit our systematic review to the philosophical-ethical debate. For inclusion, the publications must explicitly state a minimal requirement of explainability or its absence for ethical permissibility, given the objective of this review. However, we acknowledge that in taking this approach we may overlook arguments in records where the ethical relevance of explainability is discussed, and therefore might contribute to the debate without providing recommendations on the requirements for explainability itself. Finally, we excluded literature that was not specific to the healthcare domain.

We conducted the literature screening using Rayyan^2^ for abstract screening and duplicate detection [16]. Given constraints on time and resources, we performed single screening. A study by Gartlehner et al. from 2020 showed that single screening may miss up to 13% of relevant records [17]. The review results were checked for plausibility and correctness by a second review author. These steps were performed independently, to reduce the risk of biases. However, we are optimistic that none of these limitations would alter the overall conclusions of this review. Furthermore, the exclusion of non-English literature may have leaded to missing relevant literature written in other languages.

As proposed by [12], we categorized the normative standards on the requirement of xAI and ad-hoc explainability made in the literature in conjunction with reasons. Additionally, we broadly summarized the required levels of explainability in the literature as relative or absolute levels.

### Terminology

The notions of *explainability*, *transparency*, *understandability*, *interpretability*, and *contestability*, their synonyms, hypernyms, and hyponyms, are frequently used to denote levels of epistemic opacity of AI-DSS. Depending on the research domain (e.g., social sciences, humanities, regulatory sciences, or computer sciences), these notions are either equivalent (and therefore interchangeable) or they are used with their own, distinct, and domain-specific meanings [11].

The philosophical foundations of these notions are by no means trivially summarized. In the literature, different types of explanations in scientific context were elaborated by philosophers of science for centuries [18, 19]. A complete overview of these definitions is out of the scope of this article. However, it is crucial to note that many alternative definitions to our following conception were proposed and that the definition of epistemic opacity and explainability is an active field of research in the philosophy of science.

To simplify the vocabulary for this review, we define *explainability* as a relative attribute: the degree of epistemic accessibility, i.e., the inverse of epistemic opacity as described by Humphrey. Humphrey defines the epistemic opacity of a computational system as the inaccessibility of its processes and attributes [7]. The ability to explain the system’s processes and attributes requires a certain degree of epistemic accessibility.

We define the explainability of the explanandum X as the ability of the explainer A to provide the recipient B with an explanation Y (explanans) of a resolution Z. Explainability may be understood as a relative attribute as the resolution Z of the explanation may vary, dependent on the complexity and transparency of the system, as well as the ability of the explainer A to provide an explanation. Thus, the explainability of an AI-DSS is an attribute describing the ability of either the AI-DSS to give explanations for its decision-making or an agent to explain the decision-making of an AI-DSS to a certain degree [20, 21].^3^

Given the plurality of conceptions of explanations, epistemic opacity, and explainability, we are not able to completely unify the terminology of the analyzed literature within the scope of this systematic review of reasons. However, we may encode the following distinctions on the degree and level of implementation of explainability, if made explicit in the corresponding record, using the outlined conceptual ground for this article.

Our definition of explainability concerns the epistemic accessibility of the processes and attributes of a computational system. *Transparency* most commonly refers to the epistemic accessibility of the AI-DSS’s attributes rather than the inner processes of a trained model. For instance, transparent AI-DSS may denote epistemic accessibility regarding the system’s architecture, training data and procedures, and performance metrics. Thus, transparency, by our definition, may be characterized as a class of levels of explainability as well. Yet, to improve readability, we refer to levels of explainability that fall within this definition as transparency in the remainder of the article.

Further, regarding the epistemic accessibility of the computational processes of an AI-DSS, we differentiate two approaches to by the level of implementation: ad-hoc and *post-hoc* explainability. AI-DSS that are explainable by design are denoted as ad-hoc (or ante-hoc) explainable. These systems are explainable to a certain degree by a lower complexity of the underlying processes. They do not require additional methods to derive insights into attributes and processes that influence the output of the model. Typical examples of these methods are for instance decision-trees or rule-based systems [22]. In contrast, AI-DSS that are not intrinsically explainable but use supplementary computational xAI methods to achieve a certain degree of explainability are denoted as post-hoc explainable [22].

**Table 1:** Characteristics of publications included in this systematic review

Features of publication	*n* (%) of publications
Publication Type	
Journal Article	44 (100)
Study type	
Philosophical discussion	44 (100)
Domain	
Sub-domain specific	8 (18)
Reproductive medicine	1 (2)
Pathology	1 (2)
Resource allocation	1 (2)
Psychiatry & Behavioral health	3 (7)
Medical Imaging	2 (5)
Not sub-domain specific	36 (82)

## Results

**Fig. 1:**
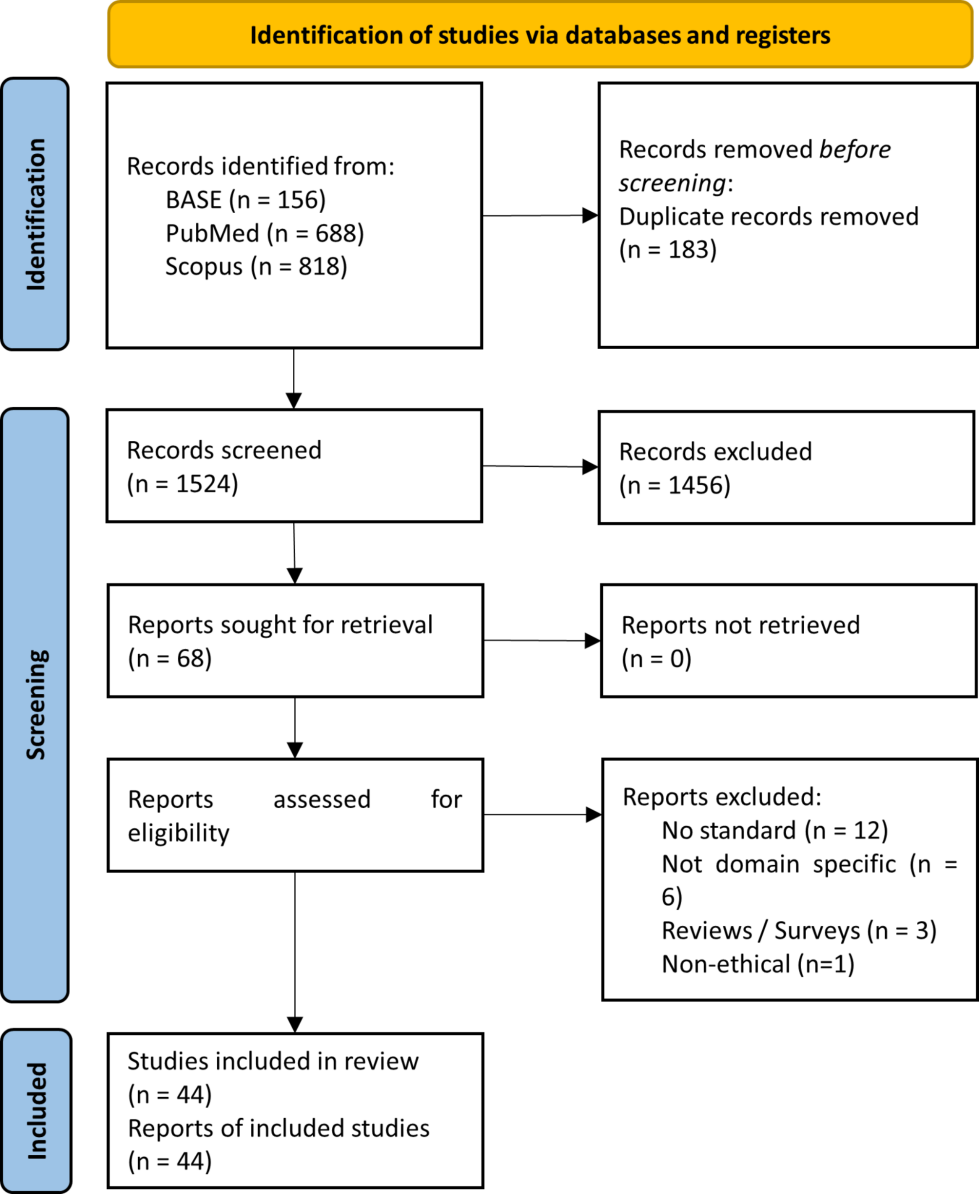
PRISMA flow chart on the literature search and screening process for publications discussing the requirement of explainability of AI-DSS in healthcare

**Fig. 2:**
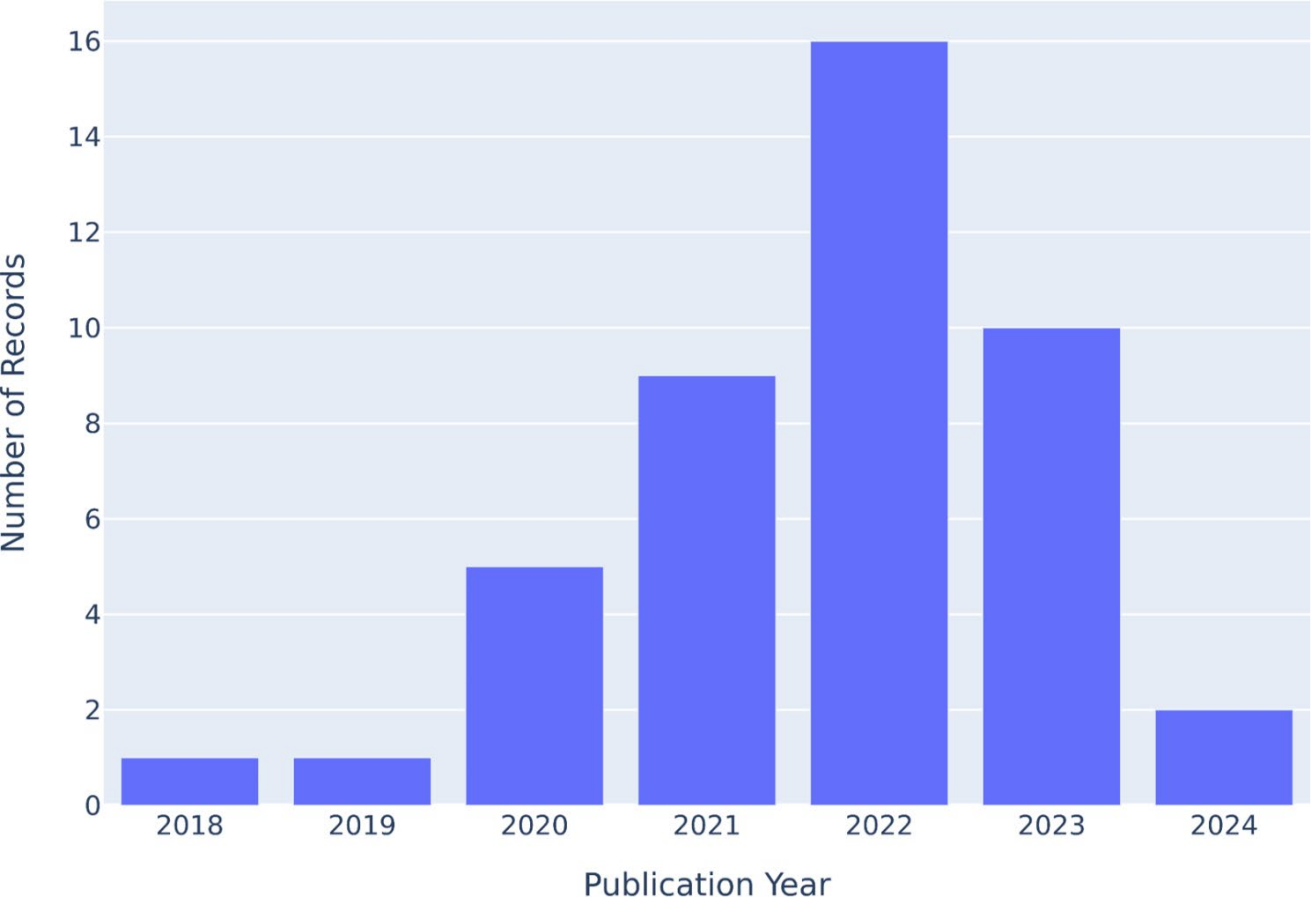
Number of records per year

The literature search yielded 1662 documents, out of which 1524 were unique. In an abstract screening, we found 68 documents to be relevant for full-text screening. We found a total of 44 documents to be relevant after assessing the full texts for eligibility. We excluded 19 records as they do not provide positions on the default requirement of explainability with respective moral reasons (non-ethical: *n* = 1; reviews: *n* = 4; no reasons: *n* = 2; no standard: *n* = 12) and 6 records that are not domain-specific (not explainability specific: *n* = 3; not healthcare specific: *n* = 2; not AI-DSS specific: *n* = 1) (cf. Figure 1). Out of 44 documents, 8 articles were specific to a sub-domain in healthcare, while the other 36 documents were generalized on AI-DSS in healthcare (cf. Table 1). The distribution of the relevant literature in publication years suggests that the discussion is not yet settled but a matter of active research, as the most prolific year we found was 2022 with 16 publications, followed by 2023 with 10 records (cf. Figure 2).

### The normative standards on the requirement of explainability

**Fig. 3:**
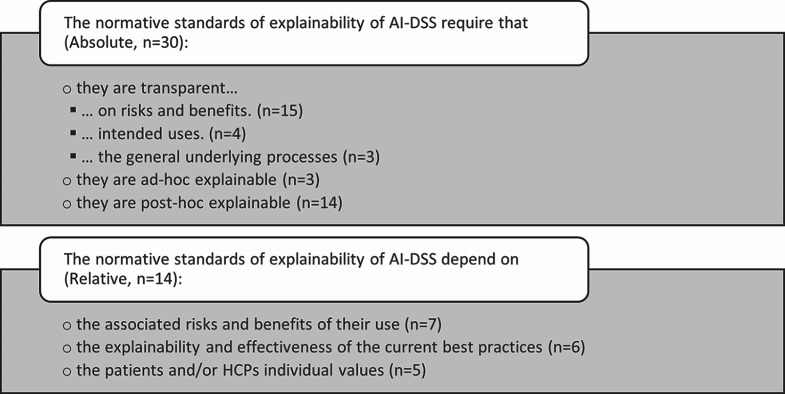
Categorization of the required levels of explainability found in the literature

Out of the 44 records, 27 argue that explainability was not required for the ethical permissibility of AI-DSS in healthcare. Therefore, based on their perspective, AI-DSS could be implemented even without providing explanations for decision-making or allowing HCPs or patients to explain their decision-making [1, 23–38], sometimes depending on the context [2, 39–47]. In contrast, we found 17 proponents of the view that explainability is a requirement for AI-DSS in healthcare [10, 48–60]. Out of these, 3 explicitly advocate for ad-hoc explainable models [51, 57, 58]. In 13 records, the authors argue that the required level of explainability depends on either the contemporary best practices [2, 39, 40], the normative reach of the decisions [40–45] and potential otherwise infeasible benefits [2, 46, 47, 61], or on the patients’ or HCPs’ values [40, 42, 61–63]. The approaches to relative normative standards of explainability can be further differentiated to dependence on risks and benefits, best practices, or patient and HCP values (cf. Figure 3). For a complete overview of the positions and their core arguments, cf. Table 2.

A major argument for the view that explainability is not a default requirement is the double standard argument. First introduced by London in 2019, the double standard argument claims that in analogy to AI-DSSs’ opaque decision-making, evidence-based medical decisions are commonly atheoretic and opaque as well [1]. London and others provide examples of pharmaceuticals and diagnostics, which find applications in healthcare but are merely empirically validated, not causally understood [1, 23–25]. Consequently, the requirement of explanations for AI-DSS would introduce double standards that require justification. Instead of explainability, Ploug & Holm demand contestability, i.e., enough information on the use of data, potential biases, performance, and the implementation of the AI-DSS in the decision-making process to reasonably contest the suggested decisions [24]. Relatedly, London, Da Silva, and McCoy et al. demand empirical evidence for the clinical performance of the AI-DSS [1, 23, 25].

In return, proponents of explainability as a default requirement assert that the comparison of AI-DSS and evidence-base medicine was flawed because the empirical evidence for the performance of AI-DSS is more likely to be confounded than for the analogies given [52] because HCPs’ decisions could be broadly explained by their social environment [48], or because other than opaque AI-DSS, HCPs could provide some useful information that that facilitate their decision-making [61].

While proponents of explainability as a default requirement insist that post-hoc xAI methods could reduce false hope and inadequate interventions [50], another epistemic presumption used as an argument against the use of post-hoc xAI methods was that those methods are a “fool’s gold” [26]. In other words, they produce a false sense of security, but in fact introduce new layers of uncertainty [30] and harbor the risk of amplifying automation biases [23, 27–29]. Interestingly, Hatherley et al., Afnan et al., and Quinn et al. agree on the flaws of xAI methods but opposingly conclude that only ad-hoc explainable models should be used for AI-DSS in healthcare [51, 57, 58].

Some also argue that less explainable models today outperform explainable models and propose a trade-off between accuracy and explainability. With an outcome-oriented perspective in favor of clinical benefits, this is a reason for some to avoid explainability as a default requirement [1, 24, 31]. However, others answer that this trade-off is only claimed but not sufficiently demonstrated [52, 58].

Advocates of explainability as a required standard list different kinds of concerns related to accountability or the attribution of responsibility. For example, Adams maintains that giving reasons is a precondition of accountability in medical decision-making [48]. Others argue that neither developers nor HCPs can be held responsible for patient harm that was a consequence of the reliance on opaque AI-DSS, resulting in a responsibility gap [51, 60]. Verdicchio and Perin note that xAI may help to recognize non-reliable suggestions and take responsible actions in turn [59]. Relatedly, Holm states that the epistemic responsibility of HCPs requires them to consider “available information and best evidence about the patient” [56], suggesting that this requires explanations from otherwise opaque AI-DSS.

Yet, opponents of that stance argue that the responsibility and accountability concerns may be addressed without the need for explainability standards. For example, in 2021 Kempt and Nagel acknowledge that non-explainable AI-DSS might implicate that disagreements between AI-DSS and HCPs may not be resolved responsibly but claim that the second opinion of another HCP may resolve this issue [33]. In a later work, Kempt et al. go one step further and argue that HCPs are merely epistemically obligated and thus responsible for taking the decision-support of beneficial AI-DSS into account but may responsibly disagree with the AI-DSS by providing reasons [32]. Similarly, Durán and Jongsma argue that HCPs are morally responsible for being justified in their actions, which may be satisfied using sufficiently reliable systems [30].

A widely accepted argument for the requirement of explainability is given by its value in finding and mitigating biases in AI-DSS [50, 52, 53, 57]. In contrast, Da Silva argues that post-hoc explainability methods could give justifications for problematic and biased decisions, thereby providing a false sense of security [23]. Moreover, while Theunissen and Browning as well as McCradden et al. acknowledge that the use of xAI methods may be valuable in the development of AI-DSS to avoid biases, in a dynamic clinical context they demand regular auditing and appropriate proxies to detect biases in practice [34, 35].

In principle, scholars from both sides acknowledge that trust, acceptance, and uptake are essential to implementing AI-DSS in healthcare. However, there are significant discrepancies in their understanding of trust.

Durán and Jongsma, with their account of computational reliabilism, take a reliabilist stance on trust: an AI-DSS is trustworthy if it is reliable [30]. Theunissen & Browning shift the burden of trustworthiness to the institution that implements the AI-DSS and require the institution to provide grounds for relying on the system [35]. Analogously, others argue that trustworthy AI-DSS require randomized clinical trials [36], interdisciplinary dialogues between developers and HCPs [36], monitoring public preferences [39], and additional technical training for HCPs [36] or users in general [37] on the general working of AI-DSS rather than explainable models [36]. McCradden et al. argue that already an understanding of potential biases and their communication may support trustworthiness [34].

In opposition, some proponents of the default requirement of explainability claim that explainability is a condition to trust recommended actions [53, 55]. Moreover, Quinn et al. address the fact that a lack of explainability may erode trust as an important validation of the model is missing [57]. Finally, it was argued that the acceptance of AI-DSS in healthcare by patients [58] or HCPs [56] requires explanations.

Ensuring autonomy, shared decision-making, and informed consent motivate predominant lines of reasons in favor of the default requirement of explainability for AI-DSS. The basic prerequisites for informed consent are (1) comprehensive and (2) understandable information. By ensuring that patients fully comprehend the implications of AI-DSS recommendations, they can actively participate in the decision-making process [10, 50]. Afnan et al., Holm, and Heinrichs and Eickhoff highlight that shared decision-making is essential for promoting patient autonomy, as it allows patients and HCPs to collaborate and reach a consensus based on the patient’s values, preferences, and clinical context. They emphasize that shared decision-making is compromised by the HCPs’ and the patients’ inability to understand AI-DSS recommendations [51, 56, 60]. Further, Herzog argues that xAI methods improve the patients’ compliance by allowing them to maintain their individual conceptions of good health and, consequently, contribute to effectiveness [63]. Obafemi-Ajayi et al. underscore the importance of the patient-HCP relationship in this context, which may also be compromised [55]. Furthermore, Afnan et al., Obafemi-Ajayi et al., and Amann et al. emphasize the value of explanations for accounting for the patients’ and HCPs’ individual values [50, 51, 55]. Finally, Riva et al. propose that explainable AI-DSS can empower both HCPs and patients to make informed decisions by enhancing their understanding of the decision-making process.

However, many advocates of the view that explainability is not a required condition for AI-DSS address shared decision-making, autonomy, and informed consent as well. In terms of trust, Theunissen and Browning argue that informed consent is not or only partially based on adequately informing about risks, precautions, or benefits but foremost on trust in the institution of medicine [35]. Herington et al. posit that complete causal explanations are not necessary and not feasible for informed consent, as only a certain level of understanding may be presupposed by the average patient [27, 28]. This is already explained by the fact that the average patient (1) is a medical layperson and (2) usually does not have an in-depth understanding of AI and digitalization. Similarly, we found the claim that transparency on risks and benefits [29, 36, 38], and specifically on data-usage, biases, and implementation [24] suffice for informed consent. Consequently, Kiener, Rueda et al., and Astromskė et al. contend that the HCPs’ duty to explain risks and benefits is satisfiable by the disclosure of risks inherited by the AI-DSS at use [36, 38], or the rationale for adopting AI-DSS in general [39], contradicting Vayena, who claims that the communication meaningful details was “fundamental tenet of medical ethics” [62].

Lastly, we found a branch of records, arguing that the requirement of explainability was context-dependent. A major argument for that position was that the more normatively far-reaching the decisions for which the recommendations of an AI-DSS are used, the higher the need for justification and, thus, explanations [40–42, 44]. Further, Ossa et al. argue that while the transparency on data origin, type, and training is almost always a required criterion, the remaining required level of explainability depends on the risk and the level of automation of the AI-DSS [43, 45]. Another perspective taken in favor of the context dependence of explainability was that the level of explanations varies on the values and capacities of the patients or HCPs [40, 42]. Finally, the point was made that potential striking benefits that could not be reached otherwise may outweigh a lack of explainability [2, 39, 46, 47]. For instance, Kempt et al. argue that, from a perspective of justice, increased accessibility of healthcare services in expert-scarce regions may justify adapting the standards of explainability to local standards until expert accessibility is equalized [2]. Relatedly [39, 46, 47], argue that a lack of alternatives may justify a lack of explainability.

**Table 2:** Core arguments for and against the default requirement of explainability

Position	Core Argument	References
The use of xAI or ad-hoc explainable models **is not a default requirement** for AI-DSS to be ethically permissible in healthcare	Medical decisions are commonly atheoretic as well (double standard argument)	[1, 23–25]
	Post-Hoc explainability methods add new levels of uncertainty and may cause false confidence	[23, 26–30]
	There can be a trade-off between accuracy and explainability	[1, 24, 31]
	Explainability is not required to resolve problems of responsibility	[30, 32, 33]
	Explainability is not required and not sufficient for the detection of biases	[23, 34, 35]
	Trust, acceptance, and uptake are feasible by transparency	[30, 34–37, 39]
	Shared decision-making, informed consent, and patient autonomy are feasible with transparency	[24, 27–29, 35, 36, 38]
	The duty of HCPs to explain risks and benefits of the medical procedures is satisfiable by transparency	[36, 38, 39]
	The associated risks of AI-DSS determine the requirement of explainability standards	[40–45]
	The capacities and values of the patients and HCPs determine the requirement of explainability standards	[40, 42]
	Potential benefits and lack of alternatives may outweigh the concerns associated with less explainable decisions	[2, 39, 46, 47]
The use of xAI or ad-hoc explainable models **is a default requirement** for AI-DSS to be ethically permissible in healthcare	The double-standard argument is an inapt comparison	[48, 52, 61]
	Explainability reduces the risk of false hope and inappropriate interventions	[50]
	The accuracy/explainability trade-off is only claimed but not substantiated	[52, 58]
	Explainability is a requirement for accountability or the attribution of responsibility	[45, 48, 51, 56, 59, 60]
	Explainability can help to find biases	[50, 52, 53, 57]
	A lack of explainability threatens trust, acceptance, and uptake	[34, 53, 55–58, 63]
	Explainability is a requirement for shared decision-making, informed consent, and patient autonomy	[10, 50, 51, 54–56, 60, 62]
	Explainability increases HCP autonomy	[54]
	Explainability is required to account for patient-values	[50, 51, 55, 63]

### The normative standards on the level of explainability

**Fig. 4:**
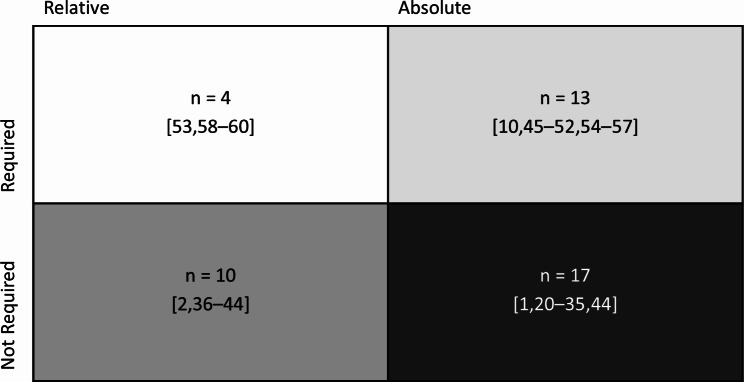
Requirement-level matrix. This matrix shows the relationship between the requirement of explainability and explainability levels

We found two broad categories of normative standards on the level of explainability and transparency in the literature: relative and absolute (cf. Figure 3). Relative standards denote explainability and transparency standards that are relative to either the associated risks or normative reach of the use of AI-DSS (cf [40–45, 56]., *n* = 7), to the effectiveness and explainability of the current best practices (cf [2, 39, 40, 46, 47, 61]., *n* = 6), or to the patients’ or HCPs’ individual values (cf [40, 42, 61–63]., *n* = 5). The absolute standards we found are supposed to generalize to the implementation of AI-DSS, independent of the context. We found requirements for the transparency on risks and benefits (cf [1, 10, 23–28, 30, 32–34, 37, 38, 53]., *n* = 15), intended uses (cf [24, 27–29]., *n* = 4), or the general underlying processes, including model-architectures or training procedures (cf [31, 35, 36]., *n* = 3). Not mutually exclusive, we found absolute standards requiring post-hoc explainable models, commonly referring to xAI methods applied to generally opaque models (cf [10, 48–50, 52–56, 59–63]., *n* = 14) and ad-hoc explainable models, referring to models that are explainable by design (e.g., decision trees) (cf [51, 57, 58]., *n* = 3). Figure 4 illustrates the relationship between the normative standards on the requirements of explainability and the level of explainability. Notably, most records (*n* = 17) position themselves in the lower right corner. This means that explainability methods and ad-hoc explainable models are not required for the ethical permissibility of AI-DSS in healthcare. Instead, many argue that statistical validation and transparency are required. Opposingly, 13 records argue that xAI or ad-hoc explainability methods are required, supported by 3 records claiming that the exact level of explainability for the xAI and ad-hoc methods should be context dependent. Finally, 10 records argue that the requirement and the level of explainability depend on the context, i.e., are relative.

## Discussion

The review of discussions on the need for post- and ad-hoc explainability in healthcare highlights ongoing debate about the requirements set by the AIA in the field of ethics. Out of 44 documents reviewed, 17 support the view that explainability is essential for the ethical use of AI-DSS in healthcare, while 27 disagree. Among the 27 that argue against the default requirement of explainability, 10 suggest that the need for explainability may depend on factors such as the level of automation, potential risks, or the established standard of care. The lack of consensus on this issue could have implications for future policymaking, particularly regarding context dependence, as the AIA currently establishes absolute required standards in the domain of healthcare. But why is there a lack of consensus? One aspect could be that the difference in technological expertise between technology producers and experts on the one hand and ethics experts on the other is becoming ever greater with increasing technical complexity. Accordingly, ethicists may find it increasingly difficult to reliably assess technical developments and systems in terms of their acceptability and normative framework conditions. This may explain why ethical assessments on the issue of explainability (but also on other issues) are currently rather disparate.

The specific level of explainability required by the standard norms needs further specification. Not all scholars explicitly recognize that explainability exists on a spectrum, such that they do not specify the degree but instead treat explainability as an absolute rather than a relative attribute [21]. Therefore, a limitation of this review is that the levels of explainability are not easily comparable and are only categorized here as requiring ad-hoc or post-hoc explanations or merely transparency^4^, absoluteness, and relativity. If a more detailed examination reveals that explainability and transparency share similar conditions, it may indicate that there is greater consensus in the literature than our findings imply.

Finally, explainability plays an important role in human-technology interaction research. In the extended theory of technology dominance, describing the effects of automation to deskilling, system transparency is taken to be an important decreasing factor [64]. Thus, further research may examine the relationship between explainability and deskilling and trigger new arguments in the ethical debate.

## Conclusions

Although there is no definitive agreement, it is evident that proponents of various requirement positions and levels of explainability frequently cite and respond to each other’s arguments. For instance, in the case of the double standard argument, but also for the value of post-hoc explainability methods or the accuracy-explainability tradeoff. Some of the disagreements arising from these arguments might be resolvable. Conducting further empirical research to compare the opacity of both healthcare professionals and AI-DSSs may help resolve disagreements on the default requirement and level of explainability. Furthermore, discussions on the quality of explanations provided by post-hoc explainability techniques and the trade-off between accuracy and explainability may need to be regularly updated, given future empirical research and the rapid technical developments in this field.

## Electronic supplementary material

Below is the link to the electronic supplementary material.

Supplementary Material 1

## Data Availability

No datasets were generated or analysed during the current study.

## Footnotes

1 Epistemology denotes the field of philosophy that examines the nature, scope, and limits of knowledge. It explores questions related to the way in which we acquire and understand information.

2 https://www.rayyan.ai/.

3 Moreover, we may differentiate *local and global explainability.* While local explainability commonly refers to the explainability of an individual decision, global explainability refers to the explainability of the system itself.

4 Transparency could also be denoted as a level of explainability (cf. Terminology).

## Abbreviations

AI: Artificial Intelligence
AIA: Artificial Intelligence Act
AI-DSS: Artificial Intelligence based Decision Support System
HCP: Healthcare Professional
xAI: Explainable Artificial Intelligence
