# Supplementary material for: The ethical requirement of explainability for AI-DSS in healthcare: a systematic review of reasons
Source: BMC Med Ethics. 2024 Oct 1;25:104. doi: 10.1186/s12910-024-01103-2 (PMC11443763; doi:10.1186/s12910-024-01103-2)
Supplement: Supplementary file 1 — Supplementary Material 1 [file 12910_2024_1103_MOESM1_ESM.docx]

**Appendices**

- 1. **Query Strings**
  2. **PubMed Query:**

“(((explainability) OR (explicability) OR (interpretability) OR (transparency) OR (contestability)) AND ((healthcare) OR (health) OR (medicine)) AND ((machine learning) OR (artificial intelligence) OR (deep learning))) AND (ethics OR (normative standards) OR (normativity))”

- 2016 – 2024

**A.2 BASE Query:**

“(explainability explicability interpretability transparency contestability) AND (healthcare health medicine) AND (“machine learning“ “artificial intelligence” “deep learning”) AND (ethics "normative standards" normativity) doctype:(11* 12* 13 14 15 19) year:[2016 TO 2024]”

- Exakte Suche
- DDC: Ethik

**A.3 Scopus Query:**

(explainability OR explicability OR interpretability OR transparency OR contestability) AND (healthcare OR health OR medicine) AND ("machine learning" OR "artificial intelligence" OR "deep learning" OR ai OR ai-dss OR ml OR dl) AND (ethics OR "normative standards") AND PUBYEAR > 2015 AND PUBYEAR < 2025 AND ( LIMIT-TO ( SUBJAREA,"ARTS" ) ) AND ( LIMIT-TO ( DOCTYPE,"ar" ) OR LIMIT-TO ( DOCTYPE,"ch" ) OR LIMIT-TO ( DOCTYPE,"cp" ) OR LIMIT-TO ( DOCTYPE,"re" ) ) AND ( LIMIT-TO ( LANGUAGE,"English" ) )
